# Peripherally Induced Regulatory T Cells: Recruited Protectors of the Central Nervous System against Autoimmune Neuroinflammation

**DOI:** 10.3389/fimmu.2017.00532

**Published:** 2017-05-09

**Authors:** Andrew Jones, Daniel Hawiger

**Affiliations:** ^1^Department of Molecular Microbiology and Immunology, Saint Louis University School of Medicine, St. Louis, MO, USA

**Keywords:** experimental autoimmune encephalomyelitis/multiple sclerosis, neuroinflammation, pTreg cells, Treg cells, tolerance, dendritic cells, CD5, HOPX

## Abstract

Defects in regulatory T cells (Treg cells) aggravate multiple sclerosis (MS) after its onset and the absence of Treg cell functions can also exacerbate the course of disease in an animal model of MS. However, autoimmune neuroinflammation in many MS models can be acutely provoked in healthy animals leading to an activation of encephalitogenic T cells despite the induction of immune tolerance in the thymus including thymically produced (t)Treg cells. In contrast, neuroinflammation can be ameliorated or even completely prevented by the antigen-specific Treg cells formed extrathymically in the peripheral immune system (pTreg cells) during tolerogenic responses to relevant neuronal antigens. This review discusses the specific roles of Treg cells in blocking neuroinflammation, examines the impact of peripheral tolerance and dendritic cells on a relevant regulation of neuroinflammation, and explores some of the most recent advances in elucidation of specific mechanisms of the conversion and function of pTreg cells including the roles of CD5 and Hopx in these processes.

## The Role of Immune Regulation in Multiple Sclerosis (MS) and Its Animal Model

During MS, immune cells attack components of the myelin sheath that surrounds the neuronal axons of the nerves of the central nervous system (CNS) leading to severe neurological symptoms. The specific autoimmune mechanisms underlying MS involve unchecked activation of autoreactive T cells (1, 2). Neuronal antigens present in the periphery may first prime the encephalitogenic T cells that subsequently migrate into the CNS where, upon re-encountering their cognate antigens, they release pro-inflammatory molecules mediating neuronal damage (1, 2). Many crucial studies on the pathogenesis and possible treatments of MS have been carried out using a mouse model of autoimmune CNS disease that in many ways mimics MS, experimental autoimmune encephalomyelitis (EAE) induced by immunization with various neuronal antigens such as myelin oligodendrocyte glycoprotein (MOG), myelin basic protein (MBP), and proteolipid protein (PLP) (3–7). The characteristic inflammation seen in EAE with perivascular CD4^+^ T cell and mononuclear cell inflammation, the clinical symptoms of progressive ascending paralysis, and the relative ease of disease induction make EAE a relevant model for MS and also a powerful model for the study of immune regulation of Th1- and Th17-dependent autoimmunity (8, 9).

Healthy individuals have myelin reactive T cells in their system, albeit, at a lower frequency than MS patients, indicating that mechanisms are in place to control these myelin-reactive T cells and prevent MS (10). Immunomodulation and Foxp3^+^ regulatory T cells (Treg cells) play a pivotal role in protection and recovery from EAE and MS by suppressing autoreactive T cells and either the absence of tTreg cells or their abnormal functions can exacerbate the severity of EAE (11–16). In addition to classically described CD4^+^CD25^+^Foxp3^+^ Treg cells, Foxp3^neg^ Tr1 cells, regulatory B cells, and CD8^+^ regulatory T cells may have various roles in regulating different aspects of the autoimmune response in EAE as discussed in Ref. (17–22). The initial observations indicating a role of Treg cells in inhibition of CNS inflammation came from experiments that showed a suppressive cell population arising during the recovery stage of EAE that was able to prevent EAE and suppress T effector cells when transferred into healthy animals (23–26). It has subsequently been shown that a transfer of *in vitro*-induced Treg cells could ameliorate EAE (27). Studies by Lafaille and colleagues showed that MBP T cell receptor (TCR) transgenic mice crossed onto a RAG-deficient background that precluded a development of Treg cells succumbed to spontaneous EAE (28). Correspondingly, a depletion of Treg cells *in vivo* by anti-CD25 antibody exacerbates EAE (29). In the early stages of MS, patients have the same frequency of Treg cells in their peripheral blood although frequencies of Treg cells are increased in the cerebrospinal fluid (CSF) of MS patients (30, 31). However, in patients suffering from MS, Treg cells may have a reduced capacity for suppression and this functional defect has been implicated in the pathogenesis of MS (30–36). Therefore, therapies focused on functions of Treg cells have been proposed as an excellent approach to block neuroinflammation some of which are reviewed in Ref. (13, 37, 38).

## Peripherally Induced Regulatory T Cells Protecting from EAE

A majority of Foxp3^+^ regulatory T cells develop in the thymus and such Treg cells are indispensable for the maintenance of immune homeostasis (39–42). However, the sudden onset of the autoimmune disease is not known to be preceded by perceivable perturbations in the functions of Treg cells despite the recognized genetic associations between T cell-related genes and MS as well as known defects in the functions of Treg cells implicated in the pathogenesis of MS (15, 43–45). Therefore, despite their crucial role in mitigation of the ongoing neuroinflammatory disease and preventing spontaneous autoimmunity in some MS models, the mechanisms dependent on thymically produced tTreg cells appear insufficient to prevent the initial priming of encephalithogenic T cells and block EAE after an immunization with relevant neuronal antigens (3, 4, 46–50). Similarly, although depletion of Treg cells inhibits spontaneous recovery from EAE, some expanding Treg cells that accumulate in CNS during EAE may not be fully efficient in controlling autoimmunity due to various reasons including possible resistance of effector T cells to Treg-mediated suppression (48, 51–53). Overall, the regulatory capacity of tTreg cells can be overwhelmed by the inflammatory injury acutely induced in healthy animals resembling the sudden onset of MS in patients.

However, EAE can be effectively prevented by the pre-administration of neuronal antigens in the non-inflammatory context. The first indication of such actively induced tolerance was provided in 1958 by a group who showed that a form of EAE could be prevented by previous administration of autoantigen in incomplete Freund’s adjuvant (IFA) (54). Further, lymph node cells transferred from rats that were treated with MBP administered without pro-inflammatory adjuvant protected recipient rats from a subsequently induced EAE (55). These early observations were then expanded in the context of the mechanisms responsible for the induction of extrathymic peripheral tolerance (56, 57). Extensive work by Stephen Miller and his co-workers showed that mouse spinal cord homogenates as well as various purified myelin derived peptides chemically coupled to splenocytes induced immune tolerance that prevented subsequently induced EAE (58–61). Additionally, tolerance preventing EAE could also be induced by microparticles that mimic apoptotic cells bearing myelin antigens (62). The T cell tolerance induced by neuronal antigenic materials relied on various immunological mechanisms including T cell anergy, however, functions of Treg cells were particularly important for the long-term maintenance of this induced tolerance (62, 63). Work by other investigators showed that treatment with tolerogenic antigens and also presentation to T cells of MOG and PLP by extrathymic dendritic cells (DCs) of the peripheral immune system could specifically prevent EAE and also increase the numbers of Treg cells (64–68). In non-EAE experimental models, DCs can convert pTreg cells *de novo* in addition to increasing the numbers and enhancing the functions of pre-existing Foxp3^+^CD25^+^ tTreg cells (69–75). Therefore, despite some clear indications of a *de novo* induction of pTreg cells, it remained unclear whether the newly converted pTreg cells were indispensible to prevent symptoms of EAE (63, 65, 67, 68, 76). Results of recent experiments using *Hopx^−/−^* mice with specific deficiencies in the functions and survival of pTreg cells but not tTreg cells helped to resolve this issue (77, 78). The severity of the EAE is the same in *Hopx^−/−^* and *Hopx^+/^*^+^ mice consistent with the unaltered functions of their tTreg cells (77, 78). However, following treatment with tolerizing myelin antigens, the *Hopx^−/−^* mice are unable to maintain long-lasting tolerance that prevents a subsequent induction of EAE (78). This defect is caused by altered suppressor functions and an increased death of *Hopx^−/−^* pTreg cells after their normal conversion from the anergic T cells following the responses to tolerogenic antigens. However, peripheral tolerance in *Hopx^−/−^* mice can be completely restored by transferred *Hopx^+/^*^+^ Foxp3^neg^ precursors that give rise to functional pTreg cells (78). In contrast, a transfer of similar numbers of pre-existing *Hopx^+/^*^+^ Foxp3^+^ tTreg cells fails to restore tolerance and prevention of EAE in *Hopx^−/−^* mice following a treatment with tolerizing myelin antigens (78). Hopx-sufficient pTreg cells are stable and maintain Foxp3 expression also under pro-inflammatory conditions (78, 79). In agreement with the crucial roles of such *de novo* differentiated pTreg cells that convert from the initially tolerized T cells, a deletion of Treg cells does not interfere with the initial induction of tolerance that depends on anergic T cells, but instead, it breaks the long-lasting maintenance of such tolerance that relies on pTreg cells that develop from the initially tolerized T cells (63, 78). Further, the induction of the pTreg cell-dependent tolerance and protection from EAE is compromised in the absence of CD5 that is required in T cells for their efficient conversion into pTreg cells (80). Similar, antigenic presentation by DCs in the absence of the pathways that increase expression of CD5 in T cells also fails to induce pTreg cells and to maintain long-lasting tolerance (79). Overall, consistent with a known division of labor between tTreg cells and pTreg cells proposed to help determine the outcomes of general auto-inflammatory responses, maternal–fetal conflict, and mucosal tolerance, pTreg cells and tTreg cells have complementary but also separate functions in regulation of neuroinflammation (75, 78, 81–84).

## Mechanisms Responsible for Induction of pTreg Cells

In contrast to pre-existing tTreg cells, antigen-specific pTreg cells first need to be converted extrathymically from the Foxp3^neg^ precursors through the mechanisms of peripheral tolerance. In the absence of this active *de novo* conversion of pTreg cells, animals remain fully susceptible to EAE similar to what is observed in mice that have a global genetic deficiency preventing either extrathymic generation of such pTreg cells or their functions and survival (75, 78). Although multiple types of APCs may have tolerogenic functions in EAE including macrophages (62, 63), DCs are particularly well equipped to regulate immune responses (85–88). In the steady state, defined by the absence of pro-inflammatory stimuli, the outcome of T cell activation by DCs results in T cell tolerance (85, 89–91). DCs first pulsed with neuronal antigens *ex vivo* and then re-injected into animals could prevent EAE similar to soluble tolerogenic antigens that can also be picked up by DCs *in vivo* (92–94). The anti-EAE tolerance is mediated by the inherent functions of endogenous DCs and an experimental induction of such tolerance was first achieved by delivering MOG *in vivo* using recombinant chimeric antibodies specific for DEC205 expressed on DCs (64). Overall, the experimental targeting to DCs or expression in DCs of MOG and other neuronal antigens has been established to induce anti-EAE tolerance (64, 66–68, 76, 78, 79). As part of their tolerogenic program, DCs induce a *de novo* induction of Foxp3 expression in extrathymic T cells converting them into pTreg cells and such pTreg cells were then also found to protect from EAE (68–72, 78, 79, 95). Tolerogenic DCs are characterized by production of various immunomodulatory metabolites and cytokines including TGF-beta and retinoic acid (70, 96–100). The tolerogenic functions of DCs can also be facilitated by the engagement of specific immunomodulatory molecules such as CTLA-4 and PD-L1 (67, 101–105). In addition to the pathways that directly affect the cell-intrinsic induction of Foxp3 expression, induction of immune tolerance and pTreg cells also depends on the specificity of T cells to self and tolerizing antigens (83, 106, 107). Such antigenic specificity is reflected by CD5, a complex regulator of T cell signaling whose expression in T cells parallels TCR signal strength during thymic selection of self-reactive T cells (108–111). Recently, CD5 was shown to instruct the extrathymic conversion of self-reactive CD5^hi^ T cells into pTreg cells by modulating their responsiveness to effector cell-differentiating cytokines through blocking the activation of the mechanistic target of rapamycin (80). In addition to thymic mechanisms, expression of CD5 in T cells can also be increased extrathymically to promote conversion of pTreg cells by tolerogenic BTLA^hi^ DCs through engagement of HVEM, a receptor for BTLA (79). Overall, CD5 increases a probability of pTreg cell conversion from T cells that have responded to either high-affinity self-peptide-MHC in the thymus or to tolerizing antigens presented by DCs of the peripheral immune system, and this mechanism may facilitate a specific formation of pTreg cells especially in the presence of pro-inflammatory cytokines (79, 80).

## The Suppressor Mechanisms of Treg Cells Relevant to Inhibition of MS and EAE

There are multiple molecular mechanisms of immune suppression by Treg cells as excellently described in Ref. (37, 112–114) and others. The Treg cells isolated from MS patients have generally been shown defective in their ability to suppress effector T cell responses and some of such Treg cells have also a decreased expression of crucial CTLA-4 as well as Foxp3 (30–36, 115, 116). In another mechanism, CD39 expressed by Treg cells removes inflammatory ATP by converting it into AMP, a substrate for CD73 that is expressed by human Th17 cells, astrocytes, and endothelial cells of the blood–brain barrier (15, 100, 117–125). Therefore, a lower frequency of CD39^+^ Treg cells in MS patients may contribute to a defective suppression in MS (119). Other surface molecules expressed by Treg cells that have been shown to modulate immune suppression include Neuropilin-1, LAG-3, TIM-3, and TIGIT (126–137). Human Treg cells also suppress effector T cells by interfering with Ca2^+^ signaling in effector T cells and this suppressor function appears to be defective in Treg cells in some MS patients as well (134, 136, 138, 139). In addition to various functional deficits, the Treg cells found in MS patients may acquire the proinflammatory phenotype of effector T cells. David Hafler’s group showed that MS patients have increased Th1 Treg cells that express IFN-γ, TBX21, and CXCR3 (140). Overall, it is clear that defects in Treg cell functions exacerbate the course of MS and could drive disease progression.

It remains unclear if the crucial molecular mechanisms of suppression differ between tTreg cells and pTreg cells. Although Hopx is expressed in both tTreg cells and pTreg cells, only pTreg cell functions and survival rely on Hopx (77, 78). The relevant molecular pathways controlled by Hopx in pTreg cells remain incompletely understood but Hopx can block expression of IL-2 in these cells (78). Treg cells, in general, rely on extracellular sources of IL-2 for their proliferation and survival and a treatment with recombinant IL-2 promotes proliferation and functions of regulatory T cells although high doses of recombinant IL-2 *in vivo* can also lead to a disappearance of Treg cell populations despite their initial expansion (53, 112, 141–148). In the absence of Hopx, pTreg cells have increased expression of the intrinsic IL-2 coinciding with their decreased suppressor ability and increased cell death. The normal functions of Hopx*^−/−^* pTreg cells can be restored by genetically ablating IL-2 expression, suggesting that increased intrinsic IL-2 expression in the absence of Hopx may be detrimental in pTreg cells (78). However, since Hopx directly modulates the expression of the AP-1 transcription complex in pTreg cells and also possibly affects other molecular pathways, the relevant complex functions of Hopx may likely be context dependent and involve other mechanisms that directly modulate pTreg cell responses (77).

## Conclusion

Studies using EAE have helped to define the pathology associated with the human disease MS and helped establish that Treg cells are vital to prevent CNS autoinflammation. Currently used therapies for MS are still not curative and often produce harmful side effects (38, 149–152). Therefore, achieving tolerance through *de novo*-induced pTreg cells that are specific for various myelin antigens might be an attractive option to effectively treat MS in addition to efforts focused on enhancing the functions of already existing Treg cells during the course of disease.

## Author Contributions

Both authors wrote and approved the final version of the manuscript.

## Conflict of Interest Statement

The authors declare that the research was conducted in the absence of any commercial or financial relationships that could be construed as a potential conflict of interest.
